# Investigating the molecular basis of local adaptation to thermal stress: population differences in gene expression across the transcriptome of the copepod *Tigriopus californicus*

**DOI:** 10.1186/1471-2148-12-170

**Published:** 2012-09-05

**Authors:** Sean D Schoville, Felipe S Barreto, Gary W Moy, Anastasia Wolff, Ronald S Burton

**Affiliations:** 1 Marine Biology Research Division, Scripps Institution of Oceanography, University of California, San Diego, 9500 Gilman Drive, La Jolla, CA, 92093-0202, U.S.A; 2 Département de Biologie, Ecole Normale Supérieure de Paris, 46, rue d'Ulm, 75005, Paris, France

**Keywords:** Next-generation sequencing, RNA-seq, Heat shock proteins, Diversification, Temperature stress, Climate

## Abstract

**Background:**

Geographic variation in the thermal environment impacts a broad range of biochemical and physiological processes and can be a major selective force leading to local population adaptation. In the intertidal copepod *Tigriopus californicus*, populations along the coast of California show differences in thermal tolerance that are consistent with adaptation, i.e., southern populations withstand thermal stresses that are lethal to northern populations. To understand the genetic basis of these physiological differences, we use an RNA-seq approach to compare genome-wide patterns of gene expression in two populations known to differ in thermal tolerance.

**Results:**

Observed differences in gene expression between the southern (San Diego) and the northern (Santa Cruz) populations included both the number of affected loci as well as the identity of these loci. However, the most pronounced differences concerned the amplitude of up-regulation of genes producing heat shock proteins (Hsps) and genes involved in ubiquitination and proteolysis. Among the *hsp* genes, orthologous pairs show markedly different thermal responses as the amplitude of *hsp* response was greatly elevated in the San Diego population, most notably in members of the *hsp*70 gene family. There was no evidence of accelerated evolution at the sequence level for *hsp* genes. Among other sets of genes, cuticle genes were up-regulated in SD but down-regulated in SC, and mitochondrial genes were down-regulated in both populations.

**Conclusions:**

Marked changes in gene expression were observed in response to acute sub-lethal thermal stress in the copepod *T. californicus*. Although some qualitative differences were observed between populations, the most pronounced differences involved the magnitude of induction of numerous *hsp* and ubiquitin genes. These differences in gene expression suggest that evolutionary divergence in the regulatory pathway(s) involved in acute temperature stress may offer at least a partial explanation of population differences in thermal tolerance observed in *Tigriopus*.

## Background

Thermal environments often vary spatially and temporally across the geographic range of a species and such variation can be an important selective force leading to genetic divergence of local populations [1]. In particular, selection resulting from the extremes in local temperature variation can have a broad impact on an organism’s genome, due to the important role of temperature on a range of biochemical and physiological processes [2]. Several studies have shown that tolerance of intertidal invertebrates to thermal stress is correlated with local temperature regimes; much of this work has focused on the tolerance limits of different species, both among unrelated taxa [3] and among congeners [4-6]. Results of these studies typically indicate that the geographic and ecological ranges of species are determined by thermal constraints, but the genetic basis for thermal tolerance is often not known and in some cases the observed phenotypic variation is driven by plasticity in traits rather than genetic differences among populations [7,8]. In order for local adaptation to evolve across environmental gradients, there must be underlying genetic variation in the phenotypic trait and moderate to strong environmental selection [9]. Additionally, rates of interpopulation gene flow must remain low, or gene swamping will override the effect of diversifying selection [10].

Several empirical studies have found evidence of the genetic fixation of alternative alleles related to local adaptation of populations along latitudinal clines [11,12] and altitudinal gradients [13,14]. These studies suggest that local adaptation sometimes involves structural changes in single protein-coding loci with large effects [15,16]. Point mutations in protein coding sequences can result in allelic gene products with differing thermal optima [17] and directly impact protein function (e.g., transporter affinity or enzyme activity) or alter the thermal stability of proteins, i.e., their propensity to lose native conformation at warmer temperatures. However, not all environmental adaptations involve changes in protein structure, as genes can alternatively evolve new patterns of expression. Natural selection on regulatory variation might play a major role in evolution of local adaptation [18,19], particularly since a single change in a master regulator gene can result in widespread changes in gene expression across the transcriptome [20]. Of course, there is also the possibility that genetic changes underlying local adaptation involve a combination of both structural changes and changes in gene regulation. Efforts to differentiate these alternatives are only now becoming feasible with next generation sequencing (permitting the analysis of regulatory elements on a genome-wide scale) and studies of transcriptome responses to the environment.

Until relatively recently, assessing structural variation of genes and gene expression during thermal stress in non-model organisms has often been limited to few genes. Heat shock proteins (Hsps) have been the most important targets for investigation because they play an important role in maintaining cellular functions under environmental stress [4,21-26]. With the recent accessibility of genomic tools for use in non-model organisms, several studies have taken advantage of microarray technology for profiling expression of hundreds to thousands of genes simultaneously. These investigations have shown that the gene-regulatory response of intertidal animals to thermal stress is much more complex than previously predicted. For instance, Lockwood et al. [5] showed that different species of *Mytilus* mussels rely, at least partially, on different sets of genes (including Hsps) during heat shock experiments. Moreover, microarray studies have revealed classes of genes that were previously unmonitored during thermal stress, but that appear to play a significant role in the thermal response [5,27-29]. Next-generation RNA sequencing technology (RNA-seq) [30] now allows us to simultaneously assemble transcriptomes and quantify gene expression across tens of thousands of genes without any *a priori* genomic information. Another useful aspect of the RNA-seq approach is that the data consist of actual transcript sequences, permitting accurate determination of expression levels of closely related paralogs in gene-rich families (such as the Hsps) that might otherwise be pooled in microarray studies [31], and identification of any structural variation among copies of ortholgous genes found in different populations.

Here we initiate investigations of the genetic mechanisms that underlie population differences in thermal tolerance observed in *Tigriopus californicus*[32]*.* Besides experiencing extreme daily and seasonal temperature fluctuations (annual range of 6-33°C at one site) in their local shallow intertidal pools [33], this species is distributed over 5000 km of coastline, from Baja California, Mexico to Alaska, spanning ~35 degrees of temperate latitude. Under moderate temperature conditions, populations of *T. californicus* show no consistent differences in survivorship [34]. Willett [32] and Kelly et al. [35] independently demonstrated that *T. californicus* exhibits pronounced interpopulation differences in tolerance to high temperature stress. With large local populations, restricted gene flow, and short generational time, we hypothesize that differences in high temperature tolerance are driven by local adaptation. As a first step in understanding the molecular mechanisms that might underlie adaptation to local thermal regimes among *T. californicus* populations, we employed RNA-seq to characterize divergence in transcriptome-wide gene expression profiles between two populations with different thermal tolerances. We additionally examine the structural variation underlying members of the *hsp70* gene family to examine whether sequence variability might account for differences between these populations.

## Methods

### Preparation of animals

At least 2000 *Tigriopus californicus* were collected from each of two natural populations, San Diego (SD: N 32.746842, W −117.254650) and Santa Cruz (SC: N 36.949589, W −122.047036), and maintained in the laboratory at 20°C with a 12-hour light:dark cycle for 1–3 generations before initiation of experiments. Two replicate samples of over 300 individual copepods of all life stages were pooled from laboratory stocks of SD and SC and flushed repeatedly in filtered (0.22 micron) seawater over two days. One sample from each population served as a control treatment, while the other sample served as the acute heat stress treatment. Pooling of large numbers of individuals into single RNA samples allows for biological averaging, and for small experimental designs, pooling has been shown to approximate non-pooled, replicated designs [36]. To initiate an acute heat stress, samples were placed in 50-ml Falcon tubes and immersed in a 35°C water bath for one hour, allowed to recover for 1 hour at 20°C, and then immediately transferred into Tri Reagent (Sigma) for RNA extraction. Samples in the control treatment were maintained at 20°C and otherwise treated identically. Samples in Tri-Reagent were disrupted with a tissue homogenizer and total RNA was then extracted following the manufacturer’s protocol.

### Illumina sequencing

The Illumina mRNA sequencing kit (RS-100-0801) and multiplexing kit (PE-400-1001) were used to isolate mRNA and prepare sequencing libraries. Matching barcodes were assigned to the SD control and heat stress sample (TGACCA), as well as the SC control and heat stress samples (GCCAAT), to allow for multiplexing on two lanes of an Illumina GA-II genome analyzer flow cell. This was done to remove possible PCR bias in comparisons of control versus heat stress samples. For sequencing, the two barcoded control samples were pooled in one lane, while the two barcoded heat stress samples pooled in an adjacent lane of the flow cell. Previous experiments have suggested that there is relatively little between-lane bias [37]. Each sample was sequenced for single-end 100 basepair read lengths.

### Preparation of reference assemblies

Reference transcriptomes for SD and SC were previously developed from 454 pyrosequencing of each population [38]. To improve coverage and representation of rare gene transcripts in these reference assemblies, we performed *de novo* assembly using the previously published 454 data and the new Illumina data. Separate assemblies were made for SD and SC sequencing reads. CLC Genomics Workbench v4.5 (CLC Bio) was used to remove adaptors and trim sequences of low quality reads, and the reads were assembled into contigs with the following assembly parameters: minimum fraction length of read overlap = 0.5, minimum sequence similarity = 0.9, minimum contig length 60 basepairs.

Assembled contigs from each population were identified as putative gene products using BLASTX searches in NCBI’s non-redundant (nr) protein database and annotated for gene function using the Blast2GO software [39,40]. The highest scoring BLAST hit, at a threshold E-value of 10^-3^, was used to assign a gene name to each assembled contig. For contigs with a positive BLAST hit, Gene Ontology (GO) [41] terms were retrieved at a threshold of 10^-6^.

Orthology between SD and SC contigs was assessed by a reciprocal best-hit approach. We used NCBI’s executable scripts to create BLAST-searchable databases with each set of contigs and then performed BLASTN searches between the two data sets. We parsed the output keeping only pairs of sequences that were each other’s best hit, with E ≤10^-20^, as putatively homologous.

### Read mapping and expression analysis

Illumina reads from SD and SC samples were mapped to their respective reference assembly using CLC Genomics Workbench (read mapping parameters: minimum fraction length of read overlap = 0.5, minimum sequence similarity = 0.99). The expression of each gene was determined by calculating the sum of the reads mapping to each contig, and then normalized by dividing the number of observed reads by the length of the contig and by the total number of reads sequenced across the transcriptome (reads per kilobase per million reads, or RPKM). To compare levels of expression between control and heat stressed samples in each population, we used Kal’s *Z*-test of proportions [42]. The *Z*-test calculates the difference in the proportion of reads observed between two treatments and compares whether each sample was drawn from the same distribution, using the normal distribution as an approximation of the binomial distribution. Due to the large number of tests, statistical significance was assessed using *p*-values adjusted by Bonferroni correction. In some cases Bonferroni can be overly conservative, so we consider tests as significant with a Bonferroni *p*-value ≤ 0.10. This was more conservative than applying a false discovery rate at *p* < 0.05, which detected many more (>10x) contigs. We then filtered the dataset of significant loci and retained only genes where at least one of the orthologous contigs had >10 mapped reads. For graphical purposes, RPKM values were log_10_ transformed due to the large range in expression values across genes. Using the GO terms for each transcriptome, we examined whether the group of genes showing a significant change in expression in each population were enriched for particular gene ontology terms relative to their reference transcriptome. Statistical significance was assessed using Fisher’s exact tests implemented in the Blast2GO software.

### Evolutionary analysis of the Hsp70 family

As discussed below, RNA-seq results identified 18 loci annotated as *hsp70*. Because these genes were prominent in the genes responsive to thermal stress, we carried out a more detailed analysis of this gene family. We performed additional BLASTX against the *Drosophila melanogaster* and *Caenorhabditis elegans* genomes in order to identify *T. californicus* contigs that were paralogous *hsp70* loci. For each identified *hsp70* copy, we aligned the orthologs between SD and SC and performed comparative analyses of sequence divergence. To look for evidence of molecular adaptation, coding sequences were compared to calculate the ratio of non-synonymous (*d*_N_) to synonymous (*d*_S_) nucleotide substitution rates [43]. Typically, *d*_N_/*d*_S_ ratios much greater than one indicate strong diversifying selection, whereas values close to one are compatible with neutral evolution and values significantly less than one are consistent with balancing or purifying selection. To examine relationships among the Hsp70 proteins, orthologs of *Drosophila melanogaster* [GenBank: NM_169441.1, NM_079632.5, NM_079615.3, NM_080059.2, NM_169469.1, NM_080188.2, NM_141952.2, NM_167306.1, NM_079017.2], *Caenorhabditis elegans* [EMBL: F26D10, C15H9, F43E2, C37H5, F44E5.4, F44E5.5, C12C8, F11F1], and *Escherichia coli* [UniProt: P77319] were obtained as out-group sequences and amino acids were aligned using the ClustalW2 algorithm [44]. A neighbor-joining tree was constructed in Mega5 [45], using the p-distances model with pairwise deletion of gaps and missing data. Support for topological relationships in the phylogram was evaluated by bootstrapping with 1,000 replicates.

### Validation of RNA-seq expression levels

In order to evaluate the accuracy of the RNA-seq expression profiles, we measured the expression of a set of heat shock genes using quantitative PCR (qPCR). We first quantified expression using the same RNA samples prepared for RNA-seq, as a means of technical replication. cDNA from the transcriptome samples was diluted to ~100 ng/ul and 1ul was amplified over 25 cycles with 200 nM of each gene specific primer (Additional file 1) using Brilliant III Ultra-Fast SYBR Green QPCR Mastermix (Agilent Technologies). Several housekeeping genes were chosen to normalize the expression of target genes, including beta-actin, glyceraldehyde 3-phosphate dehydrogenase, tubulin, and myosin. Ct values for each sample were normalized by geometric averaging of the housekeeping genes, implemented in the program GeNorm [46].

We assessed biological accuracy further by replicating the thermal stress experiment with additional samples (biological replicates). For each replicate, thirty individuals were placed in 15-mL tubes containing filtered seawater, and the experimental thermal stress was repeated as above. RNA was extracted using Tri-Reagent, with tissue homogenization performed using a Mini-BeadBeater 8 (BioSpec) with silica beads (0.175 mg) at full speed for 30s. RNA was quantified by spectrophotometry, and 500 ng of RNA were used for cDNA synthesis with the High Capacity RNA-to-cDNA kit (Applied Biosystems). The experiment was performed with three replicates per temperature for each population. Quantitative PCR was performed in 20-μL reactions with the iTaq Fast SYBR Green Supermix (Bio-Rad), and contained 2 μL of a 1:10 dilution of cDNA and 250 nM of each primer. Fluorescence was measured in a Stratagene Mx 3000P System (Stratagene), with the following thermal profile: 95°C for 3 min, followed by 40 cycles of 95°C for 10 s and 59°C for 30 s. At the end of thermal cycling, a melting curve analysis was performed to confirm the presence of single amplification products. A total of 14 target genes were surveyed, and their expression levels were normalized to the expression of housekeeping genes.

## Results

### Illumina sequencing, de novo assembly, and RNA-seq mapping

The sequencing data from this study have been submitted to the NCBI Gene Expression Omnibus under accession no. GSE38546. Single-end 100 basepair Illumina sequencing of the SD samples resulted in 9,292,573 reads, with 6,794,534 reads in the heat stress sample and 2,498,039 reads in the control sample. Sequencing of the SC samples yielded 10,794,996 reads, with 6,205,028 reads in the heat stress sample and 3,256,758 in the control sample. We combined samples from each population with previous 454 sequencing data [38] and assembled the reads into 66,943 unique SD contigs and 60,648 unique SC contigs. The average length of the SD contigs was 498 basepairs (range: 49–9,830 bp) and the average length of the SC contigs was 520 bp (range: 36–9,003 bp). These contigs formed the reference transcriptomes used in our RNA-seq gene expression analysis. Approximately 29% and 33% of the SD and SC assemblies, respectively, were annotated using BLAST, and a total of 19,622 contigs were identified as orthologs based on reciprocal BLAST to each transcriptome. Approximately 78% of the total reads were uniquely mapped in the SD control sample, while ~82% reads were uniquely mapped in the SD heat stress sample. In the SC control sample, ~71% reads were uniquely mapped, while ~80% reads were uniquely mapped in the SC heat stress sample. A distribution of the RPKM values shows that most of the contigs in each treatment had values around 10 RPKM, with slightly lower values in the heat stress samples (see Additional file 2).

### Differential gene expression

In the comparison of SD control and heat shock samples, a total of 590 contigs (0.88% of 66,943 contigs) showed evidence of a statistically significant difference in expression (Bonferroni corrected *p* <0.10). Of these 590 contigs, 234 were removed due to a low number of mapped reads, leaving 356 (0.53%) as significantly up or down regulated. In the comparison of SC control and heat shock samples, a total of 903 contigs (1.49% of 60,648 contigs) showed evidence of a statistically significant difference in expression (Bonferroni corrected *p* <0.10). Of these 903 contigs, 36 were removed due to a low number of mapped reads, leaving 867 (1.43%) as significantly up or down regulated. Most of the contigs identified as significant had RPKM values between 10 and 100, with better coverage than the overall transcriptome (see Additional file 2).

**Figure 1:**
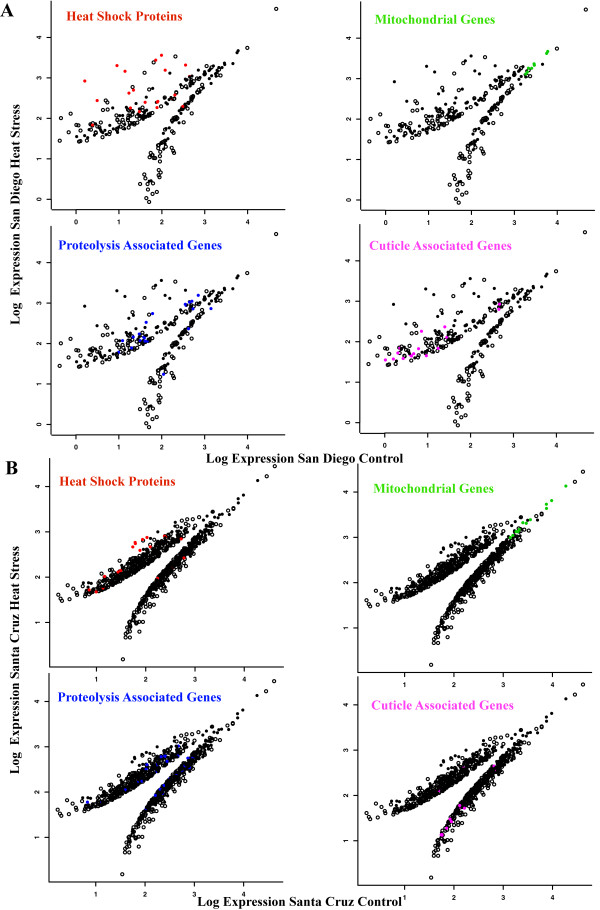
**Statistically significant up/down-regulated genes in A) San Diego and B) Santa Cruz heat stress treatments.** Scatterplots of **A)** San Diego Control versus San Diego Heat Stress and **B)** Santa Cruz Control versus Santa Cruz Heat Stress. Expression values are normalized for contig length and per million reads and log-transformed. Open circles are unidentified genes, closed black circles are BLAST-classified genes, and closed colored circles represent specific groups of classified genes.

We examined the functional classes of genes that showed the significant changes in expression and tested for enrichment of gene ontology terms relative to their frequency in each transcriptome. Among the gene ontology terms that were significantly enriched (Table 1), genes responding to stress and genes involved in cuticle formation were the most overrepresented in SD and ribosomal genes and lipid transport genes were the most overrepresented in the SC population. We also focused on the pattern of expression in four classes of genes that showed significant fold changes in both populations: heat shock proteins, genes associated with proteolysis, mitochondrial genes, and genes associated with cuticle formation (see Additional file 3 for frequency of other gene ontology terms). We plotted the log-transformed expression of genes that were significantly up or down-regulated in the heat-shock treatment relative to control samples (Figure 1). Plots of the SD heat stress versus control sample (Figure 1A) show that variation in up-regulated gene expression was greater than in down-regulated genes, with a group of 125 genes exhibiting a >10X-fold up-regulation relative to the control sample. Thirteen genes with >10X-fold up-regulation were identified as heat shock proteins (Hsps). Nineteen genes involved in the degradation of damaged proteins (proteolysis associated genes) were up-regulated, as were 20 genes involved in cuticle formation. Down-regulated genes include eight mtDNA-encoded genes involved in cellular respiration, as well as some other classes of genes (e.g. lipid transport and hydrolases, not shown). In comparison, plots of the SC heat stress versus control sample (Figure 1B) show relatively even distribution of up-regulated and down-regulated genes, with much less variation in expression than the SD treatment. Only 15 genes show a >10X-fold up-regulation, and two of these are *hsps*. Seventeen proteolytic genes were up-regulated and eighteen were down-regulated in the SC comparison. Thirteen mitochondrial genes in SC were down-regulated and show roughly the same response as in SD. In striking contrast to the SD comparison, thirteen SC genes involved in cuticle formation were largely down-regulated.

**Table 1:** Enrichment of gene ontology (GO) terms in genes that were differentially expressed during heat stress

**Enriched in SD**				
**GO-ID**	**Category: Term**	% sequences within group*		**FDR*P*-value**
		**differentially expressed**	**non-changing**	
0006950	P: response to stress	**24.07**	**2.47**	3.94 x 10^-5^
0042302	F: structural constituent of cuticle	**9.84**	**0.19**	3.94 x 10^-5^
0008199	F: ferric iron binding	**4.69**	**0.01**	0.00592
0006826	P: iron ion transport	**4.69**	**0.03**	0.00630
0006879	P: cellular iron ion homeostasis	**4.69**	**0.03**	0.00630
0005524	F: ATP binding	**31.37**	**7.52**	0.00880
0051093	P: negative regulation of developmental process	**8.06**	**0.48**	0.01300
**Enriched in SC**				
**GO-ID**	**Category: Term**	% sequences within group*^#^		**FDR*P*-value**
		**differentially expressed**	**non-changing**	
0005319	F: lipid transporter activity	**4.46**	**0.11**	1.27 x 10^-8^
0006869	P: lipid transport	**4.46**	**0.23**	1.50 x 10^-6^
0033279	C: ribosomal subunit	**6.36**	**1.24**	7.46 x 10^-4^
0032982	C: myosin filament	**2.63**	**0.25**	0.01390
0008199	F: ferric iron binding	**1.74**	**0.07**	0.01914
0006879	P: cellular iron ion homeostasis	**1.74**	**0.08**	0.02918
0006414	P: translational elongation	**3.54**	**0.62**	0.03517
0003677	F: DNA binding	*0.86*	*6.18*	0.03977
0006826	P: iron ion transport	**1.74**	**0.10**	0.03977
0009001	F: serine O-acetyltransferase activity	**1.30**	**0.03**	0.04109
0031224	C: intrinsic to membrane	*3.08*	*10.22*	0.04240

Bold and italic fonts indicate terms that were over- or under-represented, respectively, in the differentially expressed category, based on false discovery rate (FDR)-corrected Fisher’s exact tests. GO categories: F = function, P = process, C = cellular component.

*Based on number of annotated genes in SD: differentially expressed – 67; non-changing – 6980.

^#^Based on number of annotated genes in SC: differentially expressed – 234; non-changing – 7289.

Although the direction of change in expression under heat stress is the same in both populations across most genes (with cuticle-associated genes being an exception), the magnitude of up- or down-regulation is markedly different. In the SD sample, several genes showed >100-fold change in expression, while the greatest change in the SC sample was 25-fold (Table 2; Figure 1). An examination of the identity of genes with statistically significant changes in expression revealed that 63 were shared in both populations (Table 2). Twenty-five of these were up-regulated in both populations, including eight heat shock proteins. In all cases, the heat shock proteins showed higher fold changes in the SD population. Fourteen genes were down-regulated in both populations, including 7 mitochondrial genes with very similar levels of change in both populations. Finally, 24 genes showed population-specific direction of responses, 12 of which belonged to two functional classes showing opposite expression profiles. Seven cuticle proteins were up-regulated in SD (mean = 16.1-fold) and down-regulated in SC (−3.1-fold), while 5 hydrolases were down-regulated in SD (−2.9-fold) and up-regulated in SC (2.4-fold) (Table 2).

**Table 2:** Genes found in both populations with statistically significant changes in expression after thermal stress

	**Fold change**				
**Contig identification**	**SD**	**SC**	**Gene Ontology annotation**	**SD Accession**	**SC Accession**
**Up-regulated in both**					
*hsp*beta-1 #2	22.9	4.5	response to stress; response to heat	JW506232	JW525782
*hsp*beta-1 #3	24.6	4.2	response to stress	JW506234	JW525781
*hsp*beta-1 #4	105.2	4.7	response to heat	JW506233	JW525780
*hsp*20 #1	38.17	3.0	response to stress; response to heat	JW519178	JW525783
*hsp*70 #1	2.5	1.33	ATP binding; response to stress; auxin biosynthetic process	JW519142	JW525772
*hsp*70 #2	224.3	7.86	mitotic cell cycle; auxin biosynthetic process; response to heat; ATP binding; response to xenobiotic stimulus; DNA replication initiation; camera- type eye morphogenesis; response to cadmium ion; negative regulation of apoptosis; nucleus	JW519350	JW526654
*hsp*70 #5	13.0	3.8	ATP binding; response to stress; auxin biosynthetic process	JW506905	JW526653
*hsp*70 #9	460.3	7.3	ATP binding; response to stress; auxin biosynthetic process	N/A	N/A
*hsp*90 #1	5.8	3.3	protein folding; cytoplasm; unfolded protein binding; ATP binding; response to stress	JW506231	JW525777
Hypothetical protein	1.3	1.4		JW506500	JW533424
Involucrin, putative	12.6	3.8	apoptosis; protein binding	JW507027	JW526798
Map kinase-interacting serine Threonine-protein kinase 1	1.7	2.3		JW507795	JW527737
Midline fasciclin	3.6	4.9		JW508028	JW527995
Polyubiquitin	2.2	2.5	viral protein processing; proteolysis; serine-type endopeptidase activity; EC 3.4.21.0	JW519561	JW535653
Transcription factor kayak	3.6	4.3	regulation of transcription, DNA-dependent; sequence- specific DNA binding; protein dimerization activity; DNA binding; transcription factor activity; nucleus	JW519889	JW538428
Ubiquitin	2.4	2.4		JW519952	JW532240
Unknown	1.1	2.1		JW513666	JW533072
Unknown	7.1	3.7		JW513718	JW529892
Unknown	174.3	25.3		JW514975	JW531801
Unknown	46.2	9.4		N/A	N/A
Unknown	1.8	1.4		N/A	N/A
Unknown	16.4	1.9		N/A	
Unknown	4.3	2		JW518153	N/A
Unknown	6.2	1.8		JW518470	JW532411
Unknown	460.3	7.3		N/A	N/A
**Down-regulated in both**					
60s ribosomal protein L14	0.79	0.58	intracellular	JW518618	JW521020
ATP synthase subunit 6	0.78	0.67	oxidation reduction; mitochondrial	DQ913891	DQ917374
Cellular retinoic acid- binding protein 2	0.61	0.49	lipid binding; binding; transporter activity; transport	JW503430	JW522628
Cytochrome oxidase subunit 3	0.816	0.69	oxidation reduction; mitochondrial	DQ913891	DQ917374
Ferritin heavy chain 1	0.48	0.49	transition metal ion binding; cellular iron ion homeostasis; oxidoreductase activity; ferric iron binding; oxidation reduction; iron ion transport	JW505400	JW524830
Ferritin middle subunit 216/heavy chain polypeptide 1	0.71	0.61	cellular iron ion homeostasis; oxidoreductase activity; ferric iron binding; oxidation reduction; iron ion transport	N/A	JW524829
NADH dehydrogenase 4L	0.73	0.67	oxidation reduction; mitochondrial	DQ913891	DQ917374
NADH dehydrogenase subunit 1	0.71	0.7	oxidation reduction; mitochondrial	DQ913891	DQ917374
NADH dehydrogenase subunit 2	0.74	0.69	oxidation reduction; mitochondrial	DQ913891	DQ917374
NADH dehydrogenase subunit 4	0.73	0.71	oxidation reduction; mitochondrial	DQ913891	DQ917374
NADH dehydrogenase subunit 5	0.85	0.73	oxidation reduction; mitochondrial	DQ913891	DQ917374
NADH dehydrogenase subunit 6	0.71	0.67	oxidation reduction; mitochondrial	DQ913891	DQ917374
Nuclear protein 1/Serine threonine-protein phosphatase	0.69	0.59	binding; DNA binding	JW508743	JW537408
Unknown	0.67	0.56		JW517373	JW531354
**Population-specific direction of response**					
3,4-dihydroxy-2- butanone-4-phosphate synthase	0.42	2.81	metal ion binding; riboflavin biosynthetic process; isomerase activity; EC 4.1.99.12	JW517629	N/A
Cell wall-associated hydrolase	0.64	1.67	hydrolase activity	JW503411	JW522621
Cell wall-associated hydrolase	0.53	3.39	hydrolase activity	JW507653	JW527565
Cell wall-associated hydrolase	0.39	2.41	hydrolase activity	JW503414	JW527566
Cell wall-associated hydrolase	0.23	1.6	hydrolase activity	JW503409	JW522614
Cell wall-associated hydrolase	0.23	2.9	hydrolase activity	N/A	JW522624
Conserved protein	0.61	2.9		JW505232	JW524634
Conserved protein	0.57	3.0		JW504216	JW523492
Cuticle protein 248	32.9	0.46	structural constituent of cuticle	JW504378	JW523655
Cuticle protein 281/502	2.3	0.3	structural constituent of cuticle	JW504361	JW523649
Cuticle protein 283/409	36.6	0.24	structural constituent of cuticle	JW504364	JW523634
Cuticle protein 541/577	11.0	0.27	structural constituent of cuticle	JW504368	JW523638
Cuticle protein 645/704	9.3	0.42	structural constituent of cuticle	JW504358	JW523633
Cuticle protein 7/414	4.6	0.32	structural constituent of cuticle	JW504393	JW523650
Ferritin chloroplast expressed	2.9	0.61	iron ion homeostasis; oxidoreductase activity; iron binding	JW519009	JW524836
Elegans protein	0.61	2.0	signal transducer activity; membrane; signal transduction; embryonic development ending in seed dormancy; protein binding; cellular component	JW505050	JW524418
Hypothetical protein	1.5	0.65		JW519182	JW526134
Hypothetical protein	0.44	3.7		JW506539	JW526192
Hypothetical protein	0.24	4.1		JW506786	JW526524
Leucine-rich protein	0.46	4.6		JW519332	JW526187
Serine acetyltransferase	0.69	3.1	acyltransferase activity	JW504201	JW537250
Unknown	4.3	0.19		N/A	N/A
Unknown	2.1	0.69		JW518261	JW532321
Unknown	2.0	0.47		JW517851	JW533144
Unknown	1.7	0.69		JW518175	JW525297

N/A = contigs for which a NCBI accession was not created because their sequences were shorter than the minimum length allowed in a Transcriptome Shotgun Assembly (TSA) submission.

When comparing transcriptional profiles of *hsps*, some population-specific responses to heat shock were also observed (Table 3). A total of 29 putatively unique *hsp* transcripts were identified as orthologs, only nine of which showed significant change in the same direction (up-regulation) in both samples. For each of 10 SC *hsp70* paralogs that can be matched to an SD ortholog, the patterns of induction during thermal stress are similar in the two populations. However, the amplitude of the stress response is markedly different, with the SD orthologs consistently showing a higher level of stress induction in the more thermally tolerant SD population. This pattern holds across other *hsp* genes, with *hsp* induction invariably higher in SD. For 13 putative *hsps*, an orthologous copy was not identified in one population through reciprocal BLAST. These genes, missing from one transcriptome, most likely reflect an incomplete transcriptome assembly; alternatively a gene may have been lost in one of the populations. These hypotheses not yet been tested via PCR amplification from genomic DNA.

**Table 3:** RNA-seq quantification of gene expression in heat shock proteins

**Contig Idenitification**	**Fold change**		**% Divergence**	***d***_**N**_**/*d***_**S**_**Ratio**	**SD Accession**	**SC Accession**
	**SD**	**SC**				
hsf	0.7	2.4	1.2	0.104	JW505571	JW525030
*hsp*beta-1 #1	1.0	0.74	2.8	0.163	JW504867	JW522977
*hsp*beta-1 #2	22.9*	4.5*	3.1	0.062	JW506232	JW525782
*hsp*beta-1 #3	24.6*	4.2*	2.8	0.262	JW506234	JW525781
*hsp*beta-1 #4	105.2*	4.7*	5.6	0.3	JW506233	JW525780
*hsp*beta-1 #5	4.9*	2.1	2.3	0.085	JW519143	JW525778
*hsp*10	0.97	0.44*	3.2	0.035	JW501904	JW520834
*hsp*16	9.8*	1.3	4	0.349	JW506236	JW525786
*hsp*20 #1	38.2*	3.0*	3.3	0.023	JW519178	JW525783
*hsp*20 #2	n/o	4.8*	-	-	n/o	N/A
*hsp*40 #1	1.9*	1.4	1.7	0	JW506223	JW525767
*hsp*40 #2	3.2*	1.8	3	0.032	JW506406	JW526009
*hsp*60	1.1	0.74	2.5	0	JW519415	JW521007
*hsp*67b2 #1	1.2	0.59*	8.7	0.029	JW506224	JW525770
*hsp*67b2 #2	0.88	0.46*	2	0.222	JW519141	JW525771
*hsc*70	1.9*	1.6	1.2	0	JW506426	JW525790
*hsp*70 #1	2.5*	1.3*	2.9	0.014	JW519142	JW525772
*hsp*70 #2	224.3*	7.9*	3.9	0.035	JW519350	JW526654
*hsp*70 #3	0.74	1.8	1.6	0	JW506218	JW525763
*hsp*70 #4	0.92	1.3	1.9	0	JW506217	JW525759
*hsp*70 #5	13.0*	3.8*	2.6	0.015	JW506905	JW526653
*hsp*70 #6	218.9*	n/o	-	-	JW506227	n/o
*hsp*70 #7	28.2*	n/o	-	-	JW506228	n/o
*hsp*70 #8	524.4*	n/o	-	-	JW506906	n/o
*hsp*70 #9	460.3*	7.3*	3.7	0	N/A	N/A
*hsp*70 #10	n/o	8.5*	-	-	n/o	N/A
*hsp*70 #11	n/o	7.3*	-	-	n/o	N/A
*hsp*70 #12	n/o	5.1*	-	-	n/o	JW525760
*hsp*70 #13	n/o	9.6*	-	-	n/o	JW525773
*hsp*70 #14	n/o	8.9*	-	-	n/o	JW525774
*hsp*70 #15	6.1*	2.6	0	0.021	JW502097	JW521081
*hsp*70 #16	2.7	1.3	3	0.043	JW506226	JW526652
*hsp*70 #17	0.99	1.8	4.2	0.061	JW506216	JW525758
*hsp*70 #18	0.92	1.3	1.8	0	JW506217	JW525759
*hsp*70 interacting protein	41.6*	n/o	-	-	N/A	n/o
*hsp*70/90 organizing protein	3.3*	0.83	2	0.023	JW506431	JW528118
*hsp*71 #1	88.7*	3.6	4.4	0	JW506229	JW533019
*hsp*71 #2	n/o	7.1*	-	-	n/o	JW525776
*hsp*90 #1	5.8*	3.3*	3.3	0.015	JW506231	JW525777
*hsp*90 #2	n/o	7.1*	-	-	n/o	N/A
*hsp*105	1.9	1.6	1.1	0	JW506426	JW525790
mtDNA *hsp*	37.0*	n/o	-	-	JW502693	n/o

Statistically significant changes indicated by an asterisk. Uncorrected percent sequence divergence and the non-synonymous (*d*_N_) to synonymous (*d*_S_) substitution ratio are shown for trimmed alignments. Gaps in the data due to missing orthologs are indicated as “n/o”.

N/A = contigs for which a NCBI accession was not created because their sequences were shorter than the minimum length allowed in a Trascriptome Shotgun Assembly (TSA) submission.

### Sequence divergence in the hsp70 gene family

**Figure 2:**
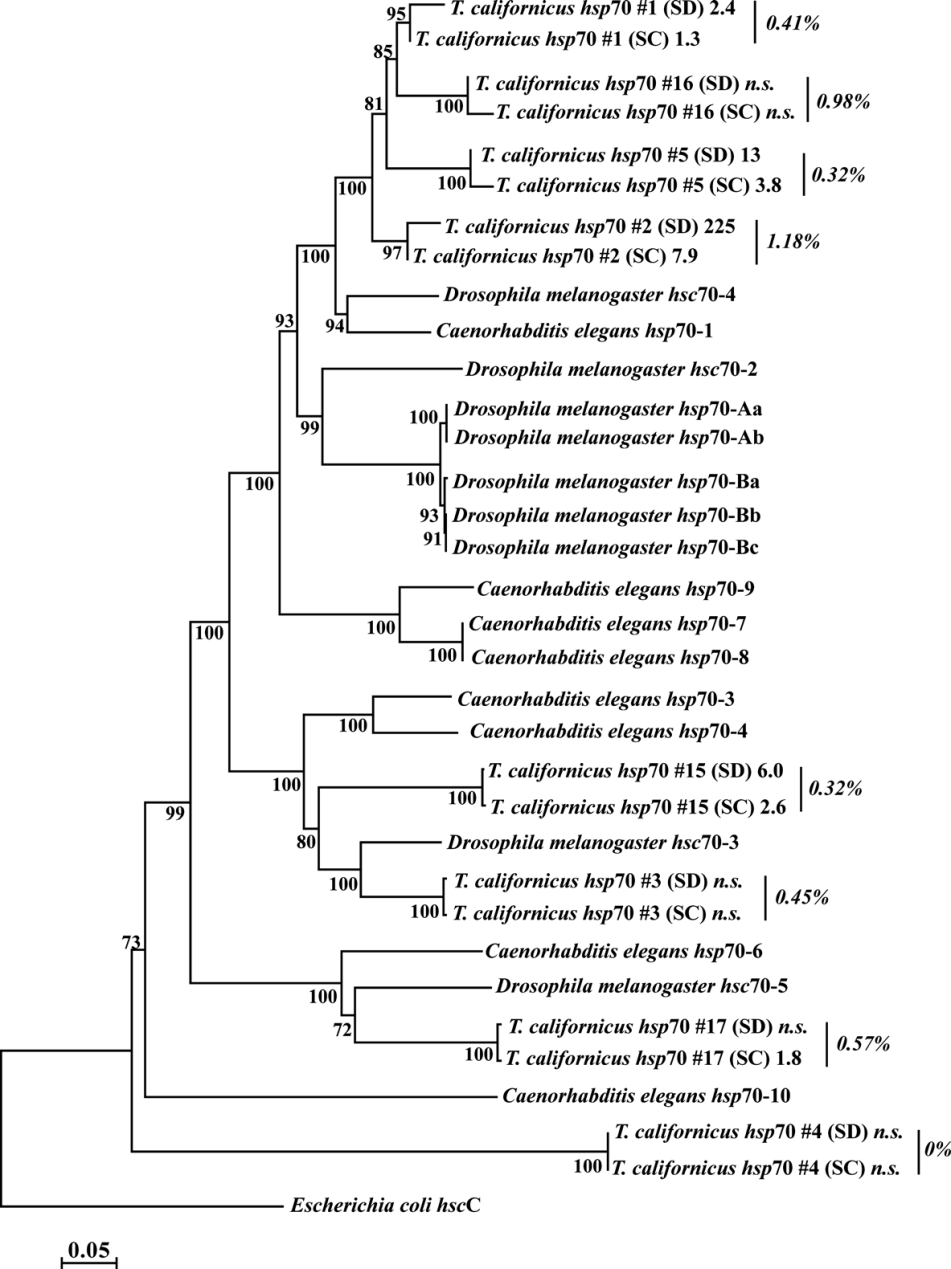
**Neighbor-joining phylogram of Hsp70 proteins from*Tigriopus californicus*and out-groups.** Numbers next to internal nodes represent bootstrap values from 1,000 replicates. For *T. californicus*, numbers next to population labels show the fold-change during thermal stress (‘n.s.’ = not significantly up- or down-regulated), as quantified by RNA-seq, while numbers associated with each pair of orthologs show the percent divergence in amino acid sequence between populations. NCBI accession numbers for *T. californicus Hsp* sequences can be found in Table 3.

Average sequence divergence of the 29 orthologous *hsp70* pairs is 2.9% and ranges from 0.0- 8.7% (Table 3). The ratio of rates of non-synonymous to synonymous substitutions (*d*_N_/*d*_S_) is less than one in all pairs, providing no evidence for divergent selection. Based on full-length transcript sequences, we unambiguously identified eight *hsp70* paralogs in the *T. californicus* transcriptome. We examined the relationship between these paralogs and Hsp70 proteins from *Drosophila melanogaster* and *Caenorhabditis elegans*. A neighbor-joining tree based on protein alignment (Figure 2) shows that five pairs have an ancient origin among Metazoans, while 3 pairs have diversified from an ancestral copy of Hsp70 #2. In all cases, orthologous copies in SD and SC are more closely related to one another than to other Hsp70 paralogs. Notably, the relatively closely related group of paralogs (#1, #2, #5, and #16) show markedly different response to thermal stress.

### Validation of RNA-seq gene expression levels

We performed qPCR assays on a subset of genes using the same RNA samples prepared for RNA-seq (a type of technical validation), as well as using RNA from additional samples (for biological validation). In nearly all comparisons (75%), fold-changes in expression measured by qPCR were in the same direction as those estimated by RNA-seq (Table 4). Moreover, the magnitude of these changes were also highly congruent between the two methods, as tested by linear regressions of log_2_ fold-differences (technical replicates: *r* = 0.85, *F*_1,34_ = 87.4, *P* < 0.001; biological replicates: *r* = 0.84, *F*_1,26_ = 68.2, *P* < 0.001).

**Table 4:** Comparison of RNA-seq and qPCR measurements of fold change in gene expression in selected genes

	**Gene**	**SD fold change**		**SC fold change**		**SD accession**	**SC accession**
		**RNA-seq**	**qPCR**	**RNA-seq**	**qPCR**		
Technical replicates	Ubiquitin	0.9	0.1	0.6	0.1	JW512238	JW538961
	*hsp*beta-1 #2	22.9	20.6	4.5	2.1	JW506232	JW525782
	*hsp*beta-1 #3	24.6	13.6	4.2	4.7	JW506234	JW525781
	*hsp*beta-1 #4	105.2	109.5	4.7	2.3	JW506233	JW525780
	*hsp*beta-1 #5	4.9	2.8	2.1	1.8	JW519143	JW525778
	*hsp*20 #1	38.2	47	3	7.1	JW519178	JW525783
	*hsp*70 #2	224	35.9	7.8	2.4	JW519350	JW526654
	*hsp*70 #3	0.7	1.1	1.8	4.5	JW506218	JW525763
	*hsp*70 #4	0.9	0.7	1.3	1.9	JW506217	JW525759
	*hsp*70 #5	13	8.2	3.8	4.2	JW506905	JW526653
	*hsp*70 #15	6.1	4.4	2.6	2.8	JW502097	JW521081
	*hsp*70 #16	2.7	1.5	1.3	1.5	JW506226	JW526652
	*hsp*70 #17	1	0.7	1.8	2.2	JW506216	JW525758
	DNAJ #1	1.1	0.5	2.1	2.5	JW503763	JW522979
	DNAJ #2	1.2	1.4	1.2	2	JW504839	JW524198
	DNAJ #3	1.2	0.7	1	4.2	JW503761	JW522978
	DNAJ #4	1.1	0.6	0.5	0.8	JW503762	JW522980
	*hsp*90 #1	5.8	1.3	3.3	7.5	JW506231	JW525777
Biological replicates	*hsp*beta-1 #3	24.6	30.7	4.2	7.3	JW506234	JW525781
	*hsp*20 #1	38.2	24.2	3	3	JW519178	JW525783
	*hsp*70 #1	2.5	11	1.3	6	JW519142	JW525772
	*hsp*70 #2	224	95.1	7.8	19.8	JW519350	JW526654
	*hsp*70 #3	0.7	1	1.8	0.6	JW506218	JW525763
	*hsp*70 #4	0.9	0.8	1.3	0.8	JW506217	JW525759
	*hsp*70 #5	13	15	3.8	5.2	JW506905	JW526653
	*hsp*70 #15	6.1	5.2	2.6	1	JW502097	JW521081
	*hsp*70 #16	2.7	1.2	1.3	1.4	JW506226	JW526652
	*hsp*70 #17	1	0.8	1.8	0.8	JW506216	JW525758
	DNAJ #1	1.1	0.9	2.1	1	JW503763	JW522979
	DNAJ #2	1.2	1	1.2	1	JW504839	JW524198
	DNAJ #3	1.2	4.2	1	2.8	JW503761	JW522978
	DNAJ #4	1.1	1.3	0.5	1.5	JW503762	JW522980

## Discussion

### Population divergence in acute heat stress

Populations of *Tigriopus californicus* show strikingly different survivorship following acute thermal stress [32], with populations in southern California (i.e. San Diego) exhibiting higher survivorship. Here we tested the hypothesis that differences in patterns of gene expression play a role in adaptation to thermal stress. Our results, based on an RNA-seq approach, demonstrated that the two populations under study have genetically diverged in their transcriptome response to acute thermal stress. While the number of reads obtained for each treatment in each population limited our power to detect rare transcripts and incomplete assemblies could result in uneven representations of genes in each population, our qPCR data confirmed that the RNAseq results are robust measurements of gene expression changes in the tested populations. Our experiment showed that statistically significant changes in expression involved a small number of genes across the transcriptome of *T. californicus* (356 in San Diego and 867 in Santa Cruz, accounting for less than 1.5% of their respective assemblies). Among the subset of genes with statistically significant changes in expression, only 63 were responsive in both populations, suggesting that the two populations have diverged in the identity of affected loci. Furthermore, population differences were evident in both the direction and magnitude of expression changes in these 63 genes. The functional groups represented by these genes also differed between the two populations, as different gene ontology terms were enriched relative to the transcriptome. The San Diego population from southern California is more tolerant of thermal stress and exhibited a much larger up-regulation of stress-related genes following heat shock. In particular, this included a large number of genes that are members of heat shock protein families. Although there is up-regulation of *hsp70* paralogs in Santa Cruz, the fold change is much smaller than observed in the San Diego population. These results contrast with numerous other studies over latitudinal gradients that have not detected interpopulation expression differences in *hsps*[26]. For example, even at the interspecific level, Lockwood et al. [5] found that the magnitude of expression of seven *hsp70* genes was strikingly similar between a northern and a southern species of *Mytilus* mussels. Only one *hsp* (*hsp24*) exhibited a strong difference in expression between the two geographically separated species.

We also examined whether structural variation in Hsp orthologs might explain differential survivorship following acute thermal stress. Sequence analyses of the Hsp gene families suggest population differentiation at the structural level is not elevated compared to the rest of the genome. We identified 42 members of Hsp families across the San Diego and Santa Cruz populations and 29 were assigned to a matching ortholog. While some differences are evident in these orthologous copies, the amount of sequence divergence (average 2.9%) is close to the median level of 2.7% sequence divergence found across all genes in a comparison of the San Diego and Santa Cruz transcriptomes [38]. The orthologous pair with the largest amount of sequence divergence in our analysis (8.7%, *hsp67b2* #1) is not an outlier considering the range of sequence divergence evident across the entire transcriptome [38]. Finally, the lack of evidence that non-synonymous substitution rates are elevated in these genes, suggests that positive selection on the coding region may have a limited role in the diversification of heat shock protein function. We note that non-synonymous substitution rates were measured across entire genes; more rigorous tests of selection based on codon-substitution models will require sampling individuals across multiple populations of *T. californicus*[47].

In addition to the Hsp genes, several other functional categories of genes showed significant response to thermal stress. One similarity between the two populations was the pattern of down-regulation of genes encoded in the mtDNA. Since certain reactions in the electron transport chain are known to be major sources of reactive oxygen species [48], a decrease in expression of mtDNA genes may represent a means of reducing stress. In contrast to the mtDNA genes, most orthologous genes showing significant change in expression in response to thermal stress have diverged either in the magnitude of expression or direction of regulation. Perhaps the most striking difference observed was in genes involved in cuticle formation; these were enriched and up-regulated in San Diego, while being down-regulated in Santa Cruz. Cuticle genes are known to be up-regulated during diapause in many invertebrates [49], presumably to reduce cuticle permeability, but their specific role during acute thermal stress is not known. Genes functioning in proteolysis, which are known to play an important role in protein degradation following stress, also showed a differential response with significant up-regulation of some genes in southern but not in northern populations of *Tigriopus.* This pattern is opposite to the pattern found in *Mytilus*[5], where northern populations exhibit an up-regulation of proteolytic genes.

In sum, our results show a variety of significant differences between populations in gene regulation following acute thermal stress and we hypothesize that these contribute to the population differences in survivorship following stress. While mRNA transcript abundance is not equivalent to protein abundance [50], other studies have shown that gene expression changes in experimental treatments are relevant predictors of organismal physiology [51], and that there is often a high correlation between gene expression and protein abundance, including the Hsp gene family [52].

### Evidence of local adaptation in *Tigriopus* californicus

Numerous species exhibit morphological and life history variation along latitudinal clines [53,54]. These traits typically involve changes in body size, phenology [11,12,55], or temperature adaptation [56]. Recently, Willett [32] and Kelly et al. [35] provided evidence that the intertidal copepod *Tigriopus californicus* exhibits variation in temperature tolerance along a latitudinal cline in California. Willett showed that populations in northern California exhibit reduced survivorship when exposed to temperatures above 36°C for one hour, while survivorship remained high in southern California populations. Additionally, southern California populations exposed to chronic temperature stress survived longer than populations from northern California. Based on observed tradeoffs in fitness across a range of temperatures, Willett argued that this provided evidence of local adaptation to temperature in *T. californicus*. Kelly et al. [35] used selection experiments to demonstrate that thermal tolerance of *T. californicus* responded to selection over 10 generations, providing direct evidence for additive genetic variation for tolerance to thermal stress. Furthermore, Kelly et al. found that total quantitative genetic variation was almost completely partitioned among populations, rather than within populations, suggesting strong local adaptation.

Our results support the conclusions of both of the above studies and provide a possible molecular basis for local adaptation, namely that the pathways that control the regulation of numerous genes involved in response to acute temperature stress have diverged. Other studies have shown that stabilizing selection often acts to prevent genetic differentiation in expression patterns among populations and among species [57,58]. The clearest pattern in our study emerges from the dramatic differences in expression in the *hsp70* gene. Heat shock genes are known to be important components of temperature adaptation and resistance to thermal stress [1,59], as they play a critical role in preventing the degradation of intracellular proteins. Whole transcriptome studies have shown that Hsps play a dominant role in heat stress response in other species [60], as well as responding to other forms of environmental stress [61,62]. Rapid adaptation to novel thermal environments by altering expression of Hsps, and that of other chaperone proteins, may be a common form of local population adaptation in diverse taxa [5].

## Conclusions

In this study we contrasted the whole genome response in gene expression between two *T. californicus* populations that exhibit differences in tolerance to thermal stress. Broad changes in gene expression were observed, with the more thermal tolerant San Diego population showing more pronounced up-regulation of *hsp* and ubiquitin pathway genes than the more sensitive Santa Cruz population. Although this up-regulation involves a large number of heat shock proteins, the most dramatic response involved the *hsp*70 gene family. Orthologous copies of the Hsps do not show evidence of divergent selection for structural changes, suggesting, at least in part, that regulatory variation between populations may play an important role in their differential sensitivity to acute heat stress.

## Competing interests

The authors’ have no competing interests to declare.

## Authors’ contributions

SDS carried conducted the heat shock treatments, carried out the transcriptome analysis, and drafted the manuscript. FSB assisted in the transcriptome assembly, qPCR analysis and helped to draft the manuscript. GWM prepared the transcriptome samples. AW sequenced the HSP genes and assisted with qPCR analysis. RSB initiated the design and coordination of the study, obtained funding, and helped to draft the manuscript. All authors read and approved the final manuscript.

## Supplementary Material

Additional file 1 **Table S1.** List of genes and primers examined in quantitative PCR trials of *Tigriopus californicus*. Click here for file

Additional file 2 **Figure S1.** Smoothed density estimates of the reads per kilobase per million reads (RPKM) of contigs in each treatment for A) the whole transcriptome and B) significantly differentially expressed genes. Click here for file

Additional file 3 **Table S2.** Gene ontology terms (F: function, P: process, C: cellular component) for significantly up or down-regulated genes. Click here for file
