# Supplementary material for: Investigating the molecular basis of local adaptation to thermal stress: population differences in gene expression across the transcriptome of the copepod Tigriopus californicus
Source: BMC Evol Biol. 2012 Sep 5;12:170. doi: 10.1186/1471-2148-12-170 (PMC3499277; doi:10.1186/1471-2148-12-170)
Supplement: Additional file 1 — Table S1. List of genes and primers examined in quantitative PCR trials of Tigriopus californicus. [file 1471-2148-12-170-S1.pdf]

Additional File 1 for Schoville et al. "Local adaption to thermal stress in *Tigriopus*"

**Table 1S**. List of genes and primers examined in quantitative PCR trials of *Tigriopus californicus*.

| Gene | Primer | 5'-3' Primer sequence |
|---|---|---|
| Beta-actin | Actin-F | CCVGCCATGTAYGTGRSCATYCARGC |
| | Actin-R | CGRTCRSCRATDCCGGGGWACATGG |
| Heat shock protein beta 1 #2 | *hsp*b1_2-F | TCCAAAGGATTGGACGGACCATGG |
| | *hsp*b1_2-R | GTTCTTCTGCTCCTTACTTCCTCG |
| Heat shock protein beta 1 #3 | *hsp*b1_3-F | CAAGAACGCCCAAGACAAGA |
| | *hsp*b1_3-R | GAGGGTCCACTGAAGAAATC |
| Heat shock protein beta 1 #4 | *hsp*b1_4-F | CTACGGATTGGAAAGATTGCGG |
| | *hsp*b1_4-R | ACGGCCTCCATTCGACAATTC |
| Heat shock protein beta 1 #5 | *hsp*b1_5-F | GCTGTGATCATCAAGATACC |
| | *hsp*b1_5-R | CATGCAATCCAACATGATGATCC |
| Heat shock protein 20 #1 | *hsp*20-F | TCGACGACGACCTTCTTATGCC |
| | *hsp*20-R | TCAAKGACATCTTGACCTCG |
| Heat shock protein 70 #1 | *hsp*70_1-F | GGGACATGAAACATTGGCCTTTC |
| | *hsp*70_1-R | CGAAATGGTCCACCAATCGGTTG |
| Heat shock protein 70 #2 | *hsp*70_2-F | CGTCTCCATCTTGTCCATTGAGG |
| | *hsp*70_2-R | ACGGCCGCACCATAGGCCACAGC |
| Heat shock protein 70 #3 | *hsp*70_3-F | CAATGGTCAAMAGAGACACRTCG |
| | *hsp*70_3-R | ACCAAGGWAAYCGGATCACG |
| Heat shock protein 70 #4 | *hsp*70_4-F | GACCTCGGTCACCCGTTGCC |
| | *hsp*70_4-R | CTCGGTCCAAAGCGTGGCGT |
| Heat shock protein 70 #5 | *hsp*70_5-F | GGAAATCATCGCCAATGACCAAGG |
| | *hsp*70_5-R | CGAATGGCACGCTTGTTAGAGCTC |
| Heat shock protein 70 #15 | *hsp*70_15-F | ATCACCATCACCAACGACAA |
| | *hsp*70_15-R | AGCCAGTCGAGCTTCTCATC |
| Heat shock protein 70 #16 | *hsp*70_16-F | CACTTGGGTGGTGAGGATTT |
| | *hsp*70_16-R | GATTTTTCCACAGGCTCCAA |
| Heat shock protein 70 #17 | *hsp*70_17-F | CGCTCGCCTTGGAACACCTTGA |
| | *hsp*70_17-R | TGGACCAATCCGGACCCAAGCA |
| Heat shock protein 90 #1 | *hsp*90-F | AGGAGCATTTGGCCGTCAAGCA |
| | *hsp*90-R | GCGGCCACGACCTCCTTGG |
| DNAJ #1 | DNAJ_1-F | GATCCAGGCCCAAAAAGAAG |
| | DNAJ_1-R | TCACATTGGCACTTCGATTC |
| DNAJ #2 | DNAJ_2-F | CGTGAAACCAGGATGGAAGT |
| | DNAJ_2-R | AAATCTCGTCGCTCAAGTCC |
| DNAJ #3 | DNAJ_3-F | AAGTGTTGGGCGATTCTGAT |
| | DNAJ_3-R | GTGAGGAACGATTGGTGGAG |
| DNAJ #4 | DNAJ_4-F | CCAACAAAGGCGACAAAGAC |
| | DNAJ_4-R | CGTCCACCTGAGCGAAAT |
| Myosin 2 light chain | Myosin-F | GTGTCGCAAAAGCAAATGAC |
| | Myosin-R | GAACCTCAACCTCCTCCTCA |
| Tubulin Alpha 1 | Tubulin-F | AGGTATGGAGGAAGGCGAAT |
| | Tubulin-R | TTCAAAGTGATGGGGAGCA |
| Ubiquitin | Ubiquitin-F | TAACGGTCAAAATGGGAAGC |
| | Ubiquitin-R | AGCAACTGGAAGATGGACGA |
| Glyceraldehyde 3-phosphate | GAPDH-F | GGAGGAGGGGATGATGTTTT |
| dehydrogenase | GAPDH-R | CAACCACGAGCAATACGAGA |
