# Supplementary material for: Investigating the molecular basis of local adaptation to thermal stress: population differences in gene expression across the transcriptome of the copepod Tigriopus californicus
Source: BMC Evol Biol. 2012 Sep 5;12:170. doi: 10.1186/1471-2148-12-170 (PMC3499277; doi:10.1186/1471-2148-12-170)
Supplement: Additional file 2 — Figure S1. Smoothed density estimates of the reads per kilobase per million reads (RPKM) of contigs in each treatment for A) the whole transcriptome and B) significantly differentially expressed genes. [file 1471-2148-12-170-S2.pdf]

Additional File 2 for Schoville et al. “Local adaption to thermal stress in *Tigriopus”*

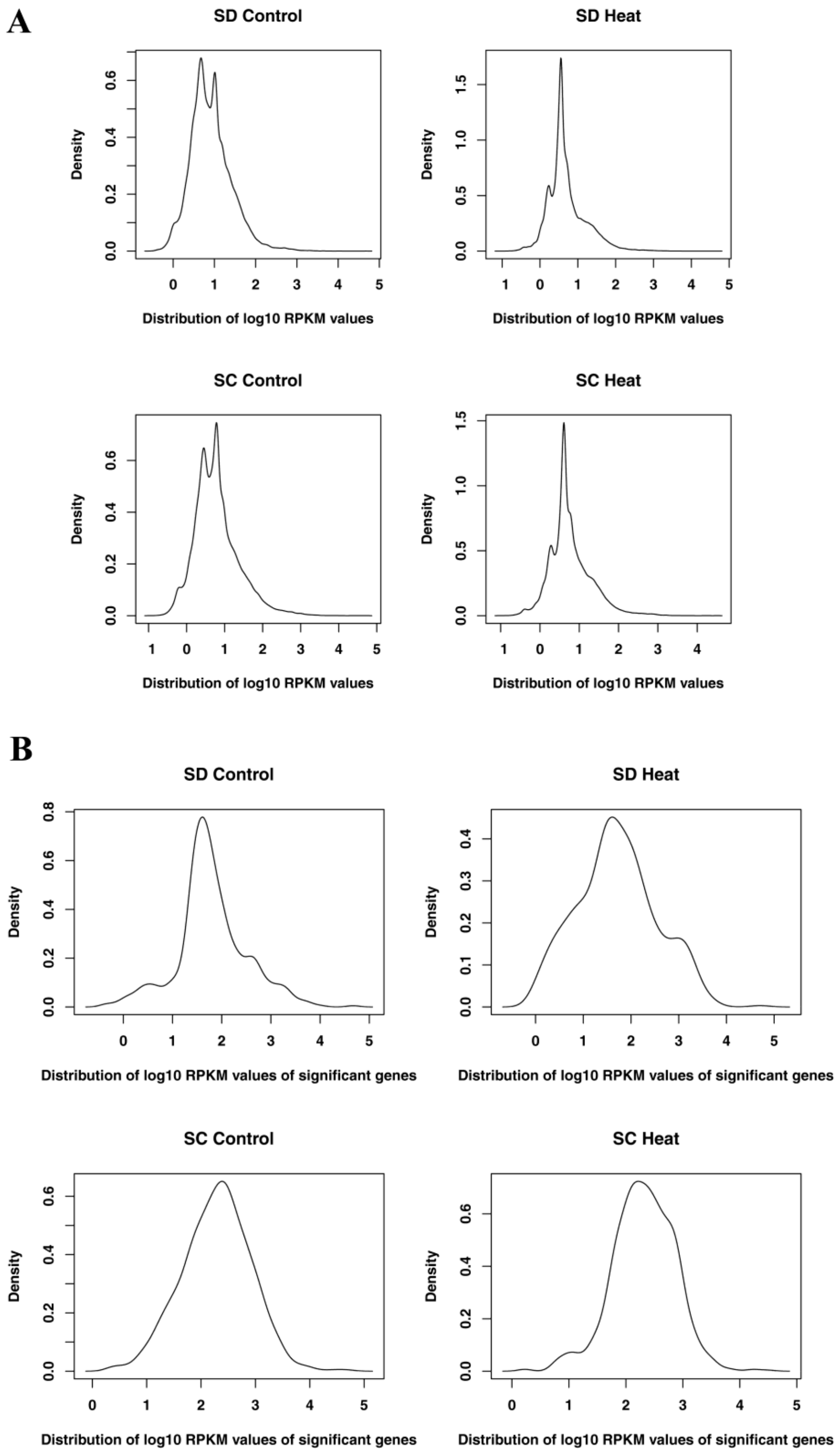

Figure 1S. Smoothed density estimates of the reads per kilobase per million reads (RPKM) of contigs in each treatment for A) the whole transcriptome and B) significantly differentially expressed genes.
