# Supplementary material for: Investigating the molecular basis of local adaptation to thermal stress: population differences in gene expression across the transcriptome of the copepod Tigriopus californicus
Source: BMC Evol Biol. 2012 Sep 5;12:170. doi: 10.1186/1471-2148-12-170 (PMC3499277; doi:10.1186/1471-2148-12-170)
Supplement: Additional file 3 — Table S2. Gene ontology terms (F: function, P: process, C: cellular component) for significantly up or down-regulated genes. [file 1471-2148-12-170-S3.pdf]

Additional File 3 for Schoville et al. “Local adaption to thermal stress in *Tigriopus”*

Table 2S. Gene ontology terms (F: function, P: process, C: cellular component) for significantly up or down-regulated genes.

**Shared GO Terms**	**SD Freq**	**SC Freq**	**San Diego Specific GO Terms**	**Freq**	**Santa Cruz Specific GO Terms**	**Freq**
C cellular_component	2	2	C caspase complex	1	C A band	2
C chloroplast	1	3	C cell cortex	1	C anchored to membrane	1
C cytoplasm	13	22	C cell surface	1	C apical cortex	2
C cytoskeleton	4	5	C endosome membrane	1	C apical plasma membrane	1
C cytosolic small ribosomal subunit	1	3	C ER-Golgi intermediate compartment	1	C cell part	2
C endoplasmic reticulum lumen	1	3	C integral to endoplasmic reticulum membrane	1	C chromosome	1
C extracellular region	5	8	C melanosome	1	C clathrin coat of coated pit	1
C integral to membrane	2	11	C membrane	1	C clathrin coat of trans-Golgi network vesicle	1
C intracellular	4	7	C mitochondrial matrix	1	C cleavage furrow	2
C lysosome	1	1	C monolayer-surrounded lipid storage body	1	C cytoplasmic part	2
C mitochondrial inner membrane	1	3	C nucleoplasm	1	C cytosolic large ribosomal subunit	1
C mitochondrial outer membrane	1	1	C plant-type cell wall	1	C eukaryotic translation initiation factor 3 complex	1
C mitochondrion	2	9	F 4 iron, 4 sulfur cluster binding	1	C extracellular space	2
C myosin complex	2	8	F acid-amino acid ligase activity	3	C focal adhesion	1
C nucleus	6	14	F aromatase activity	1	C Golgi apparatus	1
C perinuclear region of cytoplasm	1	1	F ATP-dependent protein binding	1	C heterotrimeric G-protein complex	1
C plastid	1	4	F caspase inhibitor activity	1	C intermediate filament	1
C respiratory chain	1	1	F chaperone binding	1	C intracellular membrane-bounded organelle	1
C ribosome	3	36	F endonuclease activity	1	C lamellipodium	1
F 3,4-dihydroxy-2-butanone-4-phosphate synthase activity	1	2	F heat shock protein binding	2	C large ribosomal subunit	5
F actin binding	1	7	F misfolded protein binding	1	C lipid particle	4
F amine oxidase activity	1	1	F oxidoreductase activity, acting on single donors with incorporation of molecular oxygen, incorporation of two atoms of oxygen	1	C membrane	9
F ATP binding	16	30	F phosphoinositide binding	1	C membrane part	1
F binding	6	20	F proline dehydrogenase activity	1	C microtubule	2
F calcium ion binding	2	9	F protein binding, bridging	1	C mitochondrial envelope	2
F catalytic activity	4	7	F protein disulfide oxidoreductase activity	1	C mitochondrial outer membrane translocase complex	1
F chitin binding	4	2	F protein transporter activity	1	C mitochondrial proton-transporting ATP synthase complex, coupling factor F(o)	2
F cytochrome-c oxidase activity	1	1	F structural constituent of cell wall	1	C mitochondrial respiratory chain complex IV	1
F DNA binding	2	3	F transcription factor binding	1	C myosin filament	12
F electron carrier activity	2	1	F transferase activity, transferring glycosyl groups	1	C plasma membrane	3
F ferric iron binding	3	4	P activation of signaling protein activity involved in unfolded protein response	1	C proteasome complex	1
F heme binding	1	1	P aerobic respiration	1	C proteasome core complex	1

F hydrolase activity	12	38	P anaphase-promoting complex-dependent proteasomal ubiquitin-dependent protein catabolic process	1	C proteasome core complex, alpha-subunit complex	2
F isomerase activity	1	4	P anti-apoptosis	2	C proteinaceous extracellular matrix	1
F Janus kinase activity	1	1	P axon guidance	1	C proton-transporting ATP synthase complex, catalytic core F(1)	2
F ligase activity	1	6	P binding of sperm to zona pellucida	2	C proton-transporting ATP synthase complex, coupling factor F(o)	1
F lipid binding	1	1	P biosynthetic process	2	C proton-transporting V-type ATPase, V0 domain	1
F lipid transporter activity	2	11	P camera-type eye morphogenesis	1	C ribonucleoprotein complex	2
F manganese ion binding	1	4	P cell morphogenesis involved in differentiation	1	C ruffle	1
F metal ion binding	3	15	P cellular metabolic process	1	C septate junction	1
F molecular_function	2	3	P cellular response to glucose starvation	1	C small ribosomal subunit	6
F motor activity	2	10	P cerebellar Purkinje cell layer development	1	C sodium potassium-exchanging ATPase complex	1
F non-membrane spanning protein tyrosine kinase activity	1	1	P cerebellum structural organization	1	C spindle microtubule	1
F nucleotide binding	3	7	P equator specification	1	C spliceosomal complex	1
F oxidoreductase activity	5	9	P ER overload response	1	C striated muscle thick filament	2
F protein binding	10	19	P ER-associated protein catabolic process	2	C tubulin complex	1
F protein dimerization activity	2	1	P glutamate biosynthetic process	1	C unconventional myosin complex	2
F protein kinase activity	1	1	P induction of apoptosis by extracellular signals	1	C Z disc	3
F protein serine/threonine kinase activity	2	1	P locomotory behavior	1	F 2 iron, 2 sulfur cluster binding	1
F receptor activity	1	6	P long-term strengthening of neuromuscular junction	1	F 2-alkenal reductase activity	1
F ribosome binding	1	1	P negative regulation of caspase activity	1	F 5S rRNA binding	1
F sequence-specific DNA binding	2	2	P negative regulation of transcription factor activity	1	F actin filament binding	1
F serine-type endopeptidase activity	3	2	P negative regulation of transforming growth factor beta receptor signaling pathway	1	F acyl-CoA dehydrogenase activity	1
F signal transducer activity	1	1	P negative regulation of ubiquitin-protein ligase activity during mitotic cell cycle	1	F acyltransferase activity	3
F structural constituent of cuticle	16	11	P penetration of zona pellucida	2	F adenosine deaminase activity	1
F structural constituent of ribosome	1	49	P peptidyl-L-beta-methylthioaspartic acid biosynthetic process from peptidyl-aspartic acid	1	F adenosylhomocysteinase activity	1
F transcription factor activity	2	1	P positive regulation of cyclin-dependent protein kinase activity during G1/S	1	F ARF guanyl-nucleotide exchange factor activity	1
F transcription regulator activity	1	2	P positive regulation of embryonic development	1	F ATPase activity, coupled	2
F transferase activity	2	5	P positive regulation of protein ubiquitination	1	F ATP-dependent helicase activity	1
F transition metal ion binding	1	1	P positive regulation of ubiquitin-protein ligase activity during mitotic cell cycle	1	F calcium sodium antiporter activity	1
F transporter activity	1	3	P proline catabolic process	1	F calcium-transporting ATPase activity	1
F unfolded protein binding	4	4	P regulation of protein folding in endoplasmic reticulum	1	F cation binding	1
F zinc ion binding	3	4	P regulation of synaptic plasticity	1	F chitin deacetylase activity	1

Additional File 3 for Schoville et al. “Local adaption to thermal stress in *Tigriopus”*

P apoptosis	1	1
P auxin biosynthetic process	11	19
P biological_process	1	3
P border follicle cell migration	1	1
P cell communication	1	1
P cell proliferation	1	1
P cell redox homeostasis	1	4
P cellular biogenic amine metabolic process	1	1
P cellular defense response	1	1
P cellular iron ion homeostasis	3	4
P cellular process	1	1
P chitin metabolic process	4	2
P compound eye photoreceptor cell differentiation	1	1
P cytokinesis	1	3
P defense response to virus	1	1
P DNA replication initiation	1	1
P electron transport chain	1	1
P embryonic development ending in seed dormancy	1	2
P encapsulation of foreign target	1	1
P eye-antennal disc morphogenesis	1	1
P germ-line stem cell division	1	1
P hemocyte proliferation	1	1
P hindgut morphogenesis	1	1
P humoral immune response	1	1
P imaginal disc-derived leg morphogenesis	1	1
P imaginal disc-derived wing morphogenesis	1	1
P intracellular protein transport	1	1
P iron ion transport	3	4
P lamellocyte differentiation	1	1
P lipid metabolic process	1	1
P lipid transport	2	11
P mitotic cell cycle	1	1
P multicellular organismal development	1	2
P multicellular organismal process	1	1
P negative regulation of apoptosis	2	2

P response to cadmium ion	1
P response to xenobiotic stimulus	1
P signaling	1
P sperm motility	2
P spermatogenesis	1
P viral protein processing	1

F cyclin binding	1
F cysteine-type endopeptidase activity	2
F DNA-(apurinic or apyrimidinic site) lyase activity	1
F double-stranded RNA binding	1
F endopeptidase inhibitor activity	2
F enzyme inhibitor activity	2
F exonuclease activity	1
F extracellular ligand-gated ion channel activity	1
F FAD binding	1
F glutathione peroxidase activity	1
F glutathione transferase activity	1
F glyceraldehyde-3-phosphate dehydrogenase (phosphorylating) activity	1
F GTP binding	7
F GTPase activity	6
F histone binding	2
F Hsp90 protein binding	1
F hydrogen ion transmembrane transporter activity	2
F hydrogen ion transporting ATP synthase activity, rotational mechanism	3
F hydrogen-exporting ATPase activity, phosphorylative mechanism	1
F hydrolase activity, acting on carbon-nitrogen (but not peptide) bonds	1
F hydrolase activity, acting on glycosyl bonds	2
F iron ion binding	2
F iron-sulfur cluster binding	1
F isocitrate dehydrogenase (NAD+) activity	2
F ligase activity	1
F L-malate dehydrogenase activity	1
F lyase activity	4
F metalloendopeptidase activity	2
F metallopeptidase activity	2
F methionine adenosyltransferase activity	2
F methyltransferase activity	1
F microfilament motor activity	2
F monovalent inorganic cation transmembrane transporter activity	1
F myosin light chain binding	2
F N-acetyltransferase activity	1

P ommatidial rotation	1	1
P open tracheal system development	1	1
P ovarian follicle cell stalk formation	1	1
P oxidation reduction	6	17
P periodic partitioning	1	1
P positive regulation of transcription	1	1
P primary sex determination	1	1
P protein amino acid autophosphorylation	1	1
P protein folding	4	8
P protein modification process	3	1
P proteolysis	3	3
P regulation of hemocyte differentiation	1	1
P regulation of transcription, DNA-dependent	2	3
P response to heat	10	5
P response to stress	14	18
P riboflavin biosynthetic process	1	2
P signal transduction	1	1
P somatic stem cell division	1	1
P sphingolipid metabolic process	1	1
P STAT protein nuclear translocation	1	1
P stem cell maintenance	1	1
P translation	1	46
P transport	4	3
P tyrosine phosphorylation of STAT protein	1	1
Unknown	200	436

F NAD or NADH binding	3
F NADH dehydrogenase (ubiquinone) activity	1
F nucleic acid binding	6
F nucleoside diphosphate kinase activity	1
F nucleotidyltransferase activity	1
F ornithine decarboxylase inhibitor activity	1
F peptidase activity	4
F peptidase inhibitor activity	2
F peptidyl-prolyl cis-trans isomerase activity	2
F peroxidase activity	3
F peroxiredoxin activity	1
F phosphoenolpyruvate carboxykinase (GTP) activity	1
F phosphorylase activity	1
F protein homodimerization activity	2
F proton-transporting ATPase activity, rotational mechanism	2
F purine nucleotide binding	1
F pyridoxal phosphate binding	1
F pyruvate dehydrogenase activity	1
F retinal dehydrogenase activity	1
F RNA binding	9
F rRNA binding	3
F selenium binding	2
F serine O-acetyltransferase activity	3
F serine-type peptidase activity	1
F sodium potassium-exchanging ATPase activity	2
F structural constituent of cytoskeleton	1
F structural constituent of eye lens	1
F structural constituent of muscle	2
F structural molecule activity	3
F sugar binding	2
F sulfuric ester hydrolase activity	1
F superoxide dismutase activity	2
F thiosulfate sulfurtransferase activity	1
F threonine-type endopeptidase activity	3
F translation elongation factor activity	4
F translation initiation factor activity	3
F triose-phosphate isomerase activity	1

F ubiquinol-cytochrome-c reductase activity	3
P protein ubiquination	1
P acetyl-CoA biosynthetic process from pyruvate	1
P actin cytoskeleton reorganization	1
P actin filament bundle assembly	1
P adult locomotory behavior	1
P ATP biosynthetic process	3
P ATP synthesis coupled proton transport	3
P axonogenesis	1
P blastocyst development	1
P calcium ion transport	2
P carbohydrate metabolic process	4
P cell cycle	1
P chromatin organization	1
P CTP biosynthetic process	1
P cuticle pattern formation	2
P cytoskeletal anchoring at plasma membrane	1
P defense response to bacterium	1
P defense response to protozoan	1
P detection of light stimulus	1
P determination of adult lifespan	1
P DNA replication	1
P dorsal closure, amnioserosa morphology change	2
P dorsal closure, leading edge cell differentiation	2
P dorsal closure, spreading of leading edge cells	2
P ecdysone-mediated induction of salivary gland cell autophagic cell death	2
P embryonic development	1
P embryonic development ending in birth or egg hatching	1
P embryonic development via the syncytial blastoderm	1
P epithelial cell migration, open tracheal system	2
P equator specification	1
P establishment of neuroblast polarity	2
P establishment of planar polarity	2
P establishment of protein localization	2
P flight behavior	1
P gluconeogenesis	1
P glycolysis	2

Additional File 3 for Schoville et al. “Local adaption to thermal stress in *Tigriopus”*

P G-protein coupled receptor protein signaling pathway	1
P growth	1
P GTP biosynthetic process	1
P head involution	2
P histone exchange	1
P imaginal disc-derived wing hair organization	2
P inductive cell migration	1
P innate immune response	1
P ion transport	1
P iron-sulfur cluster assembly	1
P malate metabolic process	1
P Malpighian tubule morphogenesis	2
P mature ribosome assembly	1
P metabolic process	10
P microtubule-based movement	3
P mitochondrial electron transport, ubiquinol to cytochrome c	2
P mitotic spindle organization	1
P monovalent inorganic cation transport	1
P muscle attachment	3
P myosin II filament assembly	2
P negative regulation of myeloid leukocyte differentiation	1
P negative regulation of nucleotide metabolic process	1
P nematode larval development	1
P neuromuscular process	1
P nuclear mRNA splicing, via spliceosome	1
P nucleoside diphosphate phosphorylation	1
P nucleosome assembly	1
P one-carbon metabolic process	3
P ovarian follicle cell migration	2
P plasma membrane ATP synthesis coupled proton transport	2
P positive regulation of cAMP biosynthetic process	1
P positive regulation of epithelial cell proliferation	1
P positive regulation of keratinocyte differentiation	1
P positive regulation of transcription from RNA polymerase II promoter	1
P potassium ion transport	1

Additional File 3 for Schoville et al. “Local adaption to thermal stress in *Tigriopus”*

P primitive hemopoiesis	1
P pronuclear migration	1
P protein homooligomerization	1
P protein oligomerization	2
P protein polymerization	3
P protein targeting	1
P protein transport	1
P protein ubiquitination	5
P proteolysis involved in cellular protein catabolic process	1
P regulation of ARF protein signal transduction	1
P regulation of cell shape	1
P regulation of proteolysis	1
P regulation of translation	1
P regulation of tube diameter, open tracheal system	1
P regulation of tube length, open tracheal system	1
P response to glucagon stimulus	1
P response to mechanical stimulus	1
P response to oxidative stress	1
P response to selenium ion	1
P response to stimulus	1
P response to temperature stimulus	1
P ribosomal small subunit biogenesis	1
P ribosome biogenesis	2
P rRNA processing	2
P sarcomere organization	2
P septate junction assembly	1
P skeletal muscle thick filament assembly	2
P small GTPase mediated signal transduction	1
P sodium ion transport	1
P striated muscle contraction	2
P sulfate transport	1
P superoxide metabolic process	2
P tissue homeostasis	1
P translational elongation	8
P translational initiation	1
P transmembrane transport	3
P tricarboxylic acid cycle	3
P tRNA processing	1

P	ubiquitin-dependent protein catabolic process	4
P	UTP biosynthetic process	1
P	vesicle-mediated transport	1
